# Barley RNA viromes in six different geographical regions in Korea

**DOI:** 10.1038/s41598-018-31671-4

**Published:** 2018-09-05

**Authors:** Yeonhwa Jo, Ju-Young Bae, Sang-Min Kim, Hoseong Choi, Bong Choon Lee, Won Kyong Cho

**Affiliations:** 1 Research Institute of Agriculture and Life Sciences, College of Agriculture and Life Sciences, Seoul National University, Seoul, 08826 Republic of Korea; 2 Crop Foundation Division, National Institute of Crop Science, RDA, Wanju, 55365 Republic of Korea

## Abstract

Barley is a kind of cereal grass belonging to the family *Poaceae*. To examine viruses infecting winter barley in Korea, we carried out a comprehensive study of barley RNA viromes using next-generation sequencing (NGS). A total of 110 barley leaf samples from 17 geographical locations were collected. NGS followed by extensive bioinformatics analyses revealed six different barley viromes: *Barley yellow mosaic virus* (BaYMV), *Barley mild mosaic virus* (BaMMV), *Barley yellow dwarf virus* (BYDV), *Hordeum vulgare endornavirus* (HvEV), and *Barley virus G* (BVG). BaYMV and HvEV were identified in all libraries, while other viruses were identified in some specific library. Based on the number of virus-associated reads, BaYMV was a dominant virus infecting winter barley in Korea causing yellow disease symptoms. We obtained nearly complete genomes of six BaYMV isolates and two BaMMV isolates. Phylogenetic analyses indicate that BaYMV and BaMMV were largely grouped based on geographical regions such as Asia and Europe. Single nucleotide polymorphisms analyses suggested that most BaYMV and BaMMV showed strong genetic variations; however, BaYMV isolate Jeonju and BaMMV isolate Gunsan exhibited a few and no SNPs, respectively, suggesting low level of genetic variation. Taken together, this is the first study of barley RNA viromes in Korea.

## Introduction

Barley (*Hordeum vulgare* L.) is a kind of cereal grass belonging to the family *Poaceae*. Since its first cultivation in temperate regions in Eurasia about 10,000 years ago, barley has been used as animal feed and fermentable materials for beer and whisky production^1^. Barley is the fourth biggest import crop, after maize, rice, and wheat, based on production. In Korea, winter barley is usually cultivated and is mostly consumed as polished barley mixed with rice; however, it can also be used as materials for bread, noodle, pastes, tea, beverage, and oils.

Barley is susceptible to diverse pathogens, including bacteria, fungi, and viruses. For example, the fungus *Pyrenophora teres* Drechsler, causing net blotch of barley, is a major disease in barley-growing regions^2^. Furthermore, soil-borne *Fusarium* species causing Fusarium head blight results in high yield losses^3^.

To date, several viruses infecting barley have been identified (Table 1). Of identified viruses infecting barley, the two closely related ones, *Barley yellow mosaic virus* (BaYMV) and *Barley mild mosaic virus* (BaMMV), which are members of the genus *Bymovirus* in the family *Potyviridae*, are well known. Both are important viruses infecting barley, causing yellow mosaic disease and leading to serious yield loss^4^. Both BaYMV and BaMMV are single-strand viruses, and their genomes are composed of two RNA segments, RNA1 and RNA2^5–8^. Both soil-borne BaYMV and BaMMV are transmitted by the fungal vector *Polymyxa graminis*^9,10^.

**Table 1:** List of known major viruses infecting barley.

Index	Name of virus	Family and Genus	Reference sequence	Size (Kb)	No. of proteins	No. of genes	Reference
1	*Barley yellow striate mosaic cytorhabdovirus*	*Rhabdoviridae*; *Cytorhabdovirus*	NC_028244.1	12.71	10	10	^24^
2	*Barley virus G*	*Luteoviridae*; *Polerovirus*	NC_029906.1	5.62	6	6	^25^
3	*Barley stripe mosaic virus*	*Virgaviridae*; *Hordeivirus*	NC_003469.1 (RNA1)	3.77	1	2	^46^
			NC_003481.1 (RNA2)	3.29	4	4	^47^
			NC_003478.1 (RNA3)	3.16	2	2	^48^
4	*Barley yellow mosaic virus*	*Potyviridae*; *Bymovirus*	NC_002990.1 (RNA1)	7.64	2	2	^49^
			NC_002991.1 (RNA2)	3.58	1	1	^49^
5	*Barley mild mosaic virus*	*Potyviridae*; *Bymovirus*	NC_003483.1 (RNA1)	7.26	2	2	^6^
			NC_003482.1 (RNA2)	3.52	1	1	^5^
6	*Barley yellow dwarf virus-PAV*	*Luteoviridae*; *Luteovirus*	NC_004750.1	5.68	8	8	^50^
7	*Hordeum mosaic virus*	*Potyviridae*; *Rymovirus*	NC_005904.1	9.46	2	2	^51^
8	*Hordeum vulgare endornavirus*	*Endornaviridae*; *Alphaendornavirus*	NC_028949.1	14.24	1	1	^23^

*Barley yellow dwarf virus* (BYDV) is a single-stranded RNA virus in the genus *Luteovirus* in the family *Luteoviridae* causing yellowing symptoms^11^. BYDV presenting in the phloem of the infected plants is transmitted by aphids, and several serotypes of BYDV are classified based on the aphid vector^12^.

*Barley stripe mosaic virus* (BSMV) in the genus *Hordeivirus* usually infects two monocot crops such as barley and wheat; however, BSMV infects more than 250 plant species^13^. In addition, BSMV based vector is widely used for virus-induced gene silencing (VIGS) in barley and wheat^14^. *Hordeum mosaic virus* (HoMV) in the genus *Rymovirus* was initially isolated from barley in Alberta in Canada and infects plants in the family *Poaceae* such as wheat, oat, and rye^15^.

Several studies to identify genes conferring resistance to two bymoviruses have been reported. For example, two resistance genes, *rym4* and *rym5*, are known to be effective against a barley yellow disease complex composed of BaMMV and BaYMV^16^. However, a previous study has identified a new BaYMV strain capable of breaking *rym4*-associated resistance in barley in Belgium^17^. In addition, The barley accession PI1963 carrying the *rym11* gene confers resistance against all European strains of barley yellow mosaic disease^18,19^.

Recent rapid advances of next-generation sequencing (NGS) facilitate the identification of novel plant viruses and assembly of viral genomes^20–22^. Several different kinds of libraries have been prepared by using small RNAs, double-stranded (ds) RNAs, mRNAs, and ribosomal RNA-depleted total RNAs for NGS. For example, complete genome sequence of *Hordeum vulgare endornavirus* (HvEV), which has a dsRNA genome, has been obtained from dsRNA extraction followed by NGS using MiSeq^23^. In addition, complete genome sequence of *Barley yellow striate mosaic virus* (BYSMV) in the genus *Cytorhabdovirus* was assembled by small RNA sequencing followed by Sanger-sequencing^24^. Moreover, RNA-sequencing followed by Sanger-sequencing revealed complete genome sequence of *Barley virus G* (BVG) in the genus *Polerovirus*^25^. Furthermore, a recent study has revealed a diversity of bymoviruses in barley in France using NGS and Sanger-sequencing methods^26^.

In order to examine viruses infecting barley in Korea, we carried out a comprehensive study of barley RNA viromes using NGS. For that, we collected 110 barley samples from 17 geographical locations in six different provinces. Samples were pooled based on the collected provinces and used for library preparation. Extensive bioinformatics analyses revealed six different barley viromes in Korea.

## Results

### Collection of barley samples and library preparation

**Figure 1:**
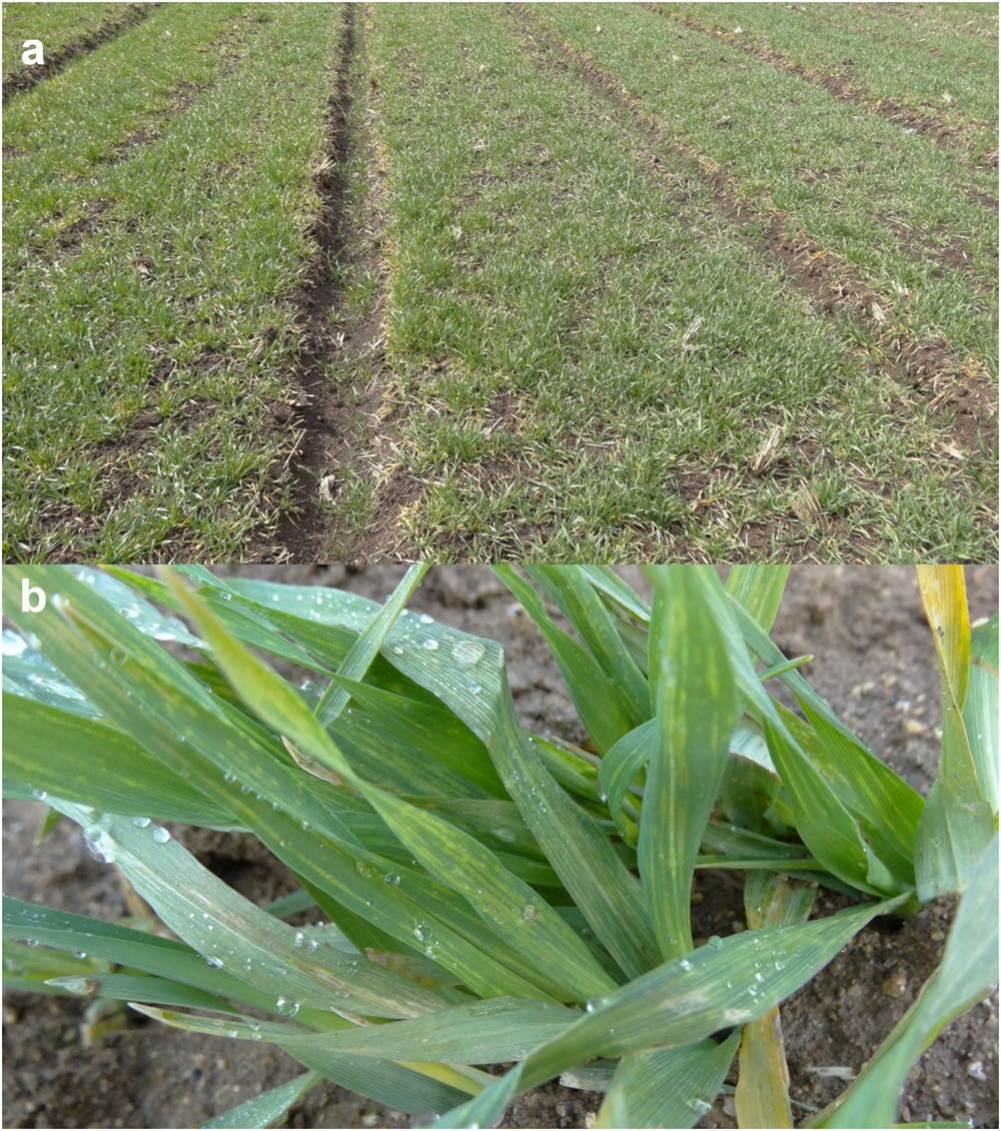
Images of barley plants showing viral disease symptoms. (**a**) The viral disease symptoms of barley plants grown in the field. (**b**) Magnified images showing yellow mosaic symptoms in barley leaves.

We collected 110 barley samples from 17 geographical locations (Table 2). Collected barley leaves showed yellow mosaic and dwarf disease symptoms (Fig. 1). Samples were pooled for total RNA extraction and library preparation based on province of collection. Six different libraries were prepared. For example, seven samples collected from Yeonggwang were assigned as library A, while 12 samples from Yeongduk and Daegu were assigned as library F. We conducted paired-end sequencing for six different libraries by HiSeq2000 system. The number of obtained reads ranged from 19,751,300 (library B) to 30,607,568 (library D) (Table 3). Obtained raw read sequences from each library were subjected to *de novo* transcriptome assembly using two different assemblers, Trinity and Velvet. The number of obtained contigs ranged from 62,074 (library B) to 250,090 (library D) by Trinity, whereas the number of contigs ranged from 997,502 (library B) to 4,103,731 (library D) (Table 4). In general, compared to Trinity, Velvet assembler produces a large number of contigs with short read length. Furthermore, the number of assembled contigs was much higher compared to other dicot plants, since the barley has a large genome.

**Table 2:** Detailed information for name of library, sample location, and number of samples in each library.

Name of library	Sample location	No. of samples
A	Yeonggwang (35.2772°N, 126.5120°E)	7
B	Gunsan (35.9677°N, 126.7366°E)	9
C	Goseong (34.9731°N, 128.3222°E), Sacheon (35.0038°N, 128.0642°E)	15
D	Jeonju (35.8242°N, 127.1480°E), Iksan (35.9483°N, 126.9576°E), Gimje (35.8036°N, 126.8809°E), Buan (35.7316°N, 126.7335°E), Gochang (35.4358°N, 126.7021°E)	25
E	Gangjin (34.6421°N, 126.7673°E), Boseong (34.7715°N, 127.0799°E), Suncheon (34.9506°N, 127.4872°E), Jangheung (34.6817°N, 126.9069°E), Haenam (34.5733°N, 126.5989°E), Yeongam (34.8002°N, 126.6968°E)	42
F	Daegu (35.8714°N, 128.6014°E), Yeongduk (36.4151°N, 129.3660°E)	12
Total	17 locations	110

**Table 3:** Summary of paired-end sequencing results for barley viromes using HiSeq2000 system.

Name of library	Total read bases (bp)	Total reads	GC (%)	AT (%)	Q20 (%)	Q30 (%)
A	2,644,932,450	26,187,450	51.085	48.91	97.105	88.537
B	1,994,881,300	19,751,300	52.12	47.88	97.087	88.441
C	3,029,794,768	29,997,968	51.779	48.22	97.086	88.455
D	3,091,364,368	30,607,568	51.86	48.14	97.158	88.672
E	2,591,080,664	25,654,264	51.039	48.96	97.219	88.88
F	2,947,953,660	29,187,660	51.665	48.34	97.169	88.695

**Table 4:** Summary of *de novo* assembly by Trinity and Velvet assemblers.

Library	A	B	C	D	E	F
Trinity	102364	62074	170521	250090	172177	108586
Velvet	1315179	997502	3003274	4103731	2993004	1569232
Total reads	26187450	19751300	29997968	30607568	25654264	29187660

Numbers indicate the number of assembled contigs by Trinity and Velvet assemblers. Total reads indicate number of all raw sequence reads in both libraries.

### Identification of viruses infecting barley

**Figure 2:**
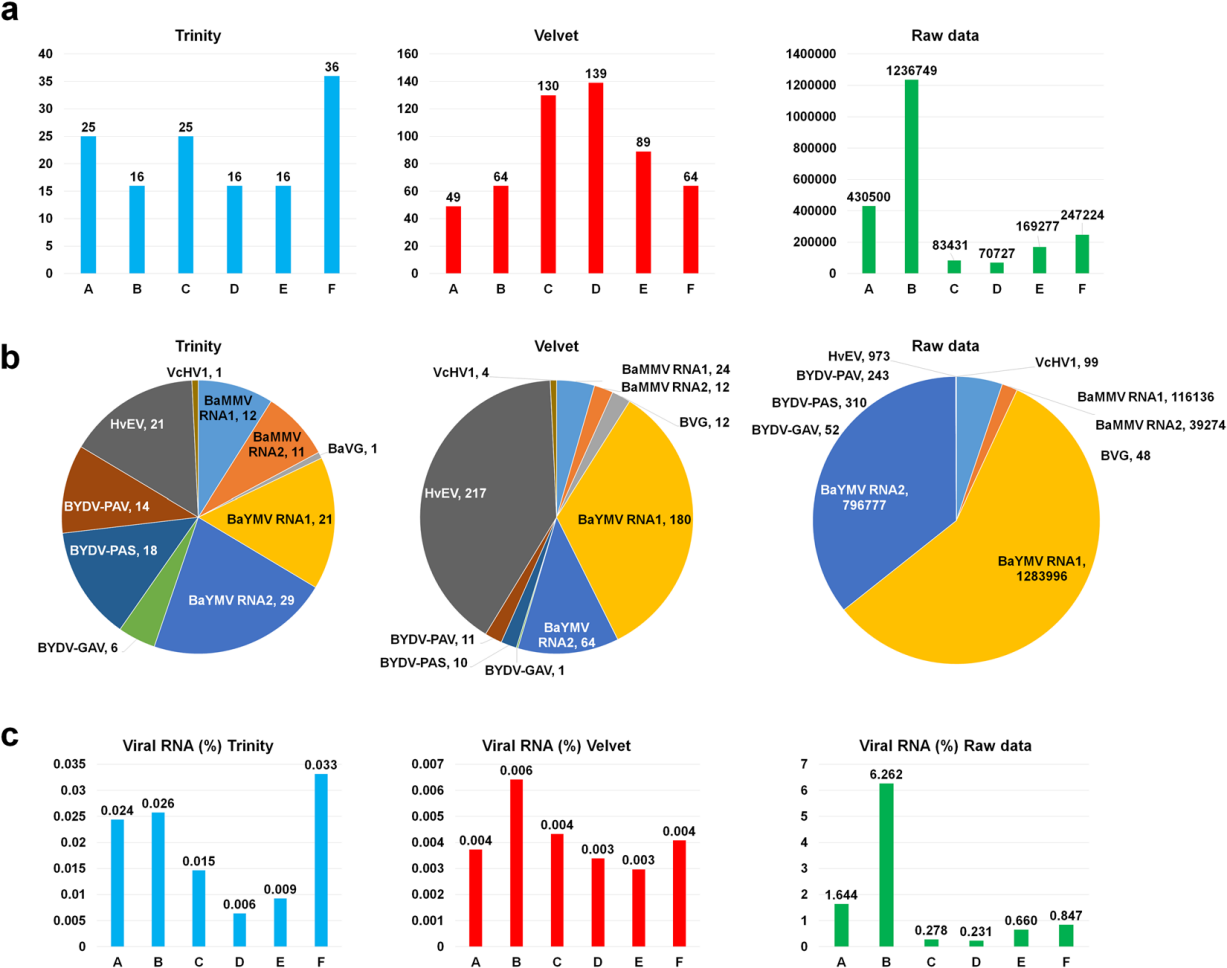
The number and proportion of virus-associated contigs and reads in each library. (**a**) The number of contigs assembled by Trinity and Velvet assemblers, and raw sequence reads associated with identified viruses in each library. (**b**) The proportion of individual viruses in all six libraries based on three different approaches, Trinity, Velvet, and raw sequence reads. (**c**) The proportion of all viral contigs and viral reads in each library.

The contigs obtained by Trinity and Velvet as well as raw sequence reads in each library were blasted against a viral reference database to identify viruses infecting barley. From contigs assembled by Trinity, the number of virus-associated contigs ranged from 16 (libraries B, D, E) to 36 (library F) (Fig. 2a and Table S1). The number of virus-associated contigs assembled by Velvet was very high as compared to those of Trinity, ranging from 49 contigs (library A) to 139 (library D). The number of raw sequence reads ranged from 70,727 (library D) to 1,236,749 (library B). The virus-associated contigs were matched to BaYMV, BaMMV, BYDV, HvEV, *Barley virus G* (BVG), and *Valsa ceratosperma hypovirus 1* (VcHV1) (Table S1). Based on number of contigs assembled by Trinity, BaYMV (50 contigs) was the dominant virus followed by BYDV (38 contigs), BaMMV (23 contigs), and HvEV (21 contigs) (Fig. 2b). In contrast, HvEV (217 contigs) was the dominant virus followed by BaYMV (244 contigs) and BaMMV (36 contigs) based on number of contigs assembled by Velvet. Based on the number of raw sequence reads, most virus-associated reads were derived from RNA1 (1,283,996 reads) and RNA2 (796,777 reads) of BaYMV.

Next, we calculated the proportion of virus-associated contigs and reads in each library (Fig. 2c). The proportion of virus-associated contigs assembled by Trinity was very low, ranging from 0.006% (library D) to 0.033% (library F), and the proportion of virus-associated contigs assembled by Velvet ranged from 0.003% (library E) and 0.006% (library B). However, the proportion of virus-associated reads in each library was increased, ranging from 0.231% (library D) to 6.262% (library B).

### Distribution of identified viruses based on geographical regions

**Figure 3:**
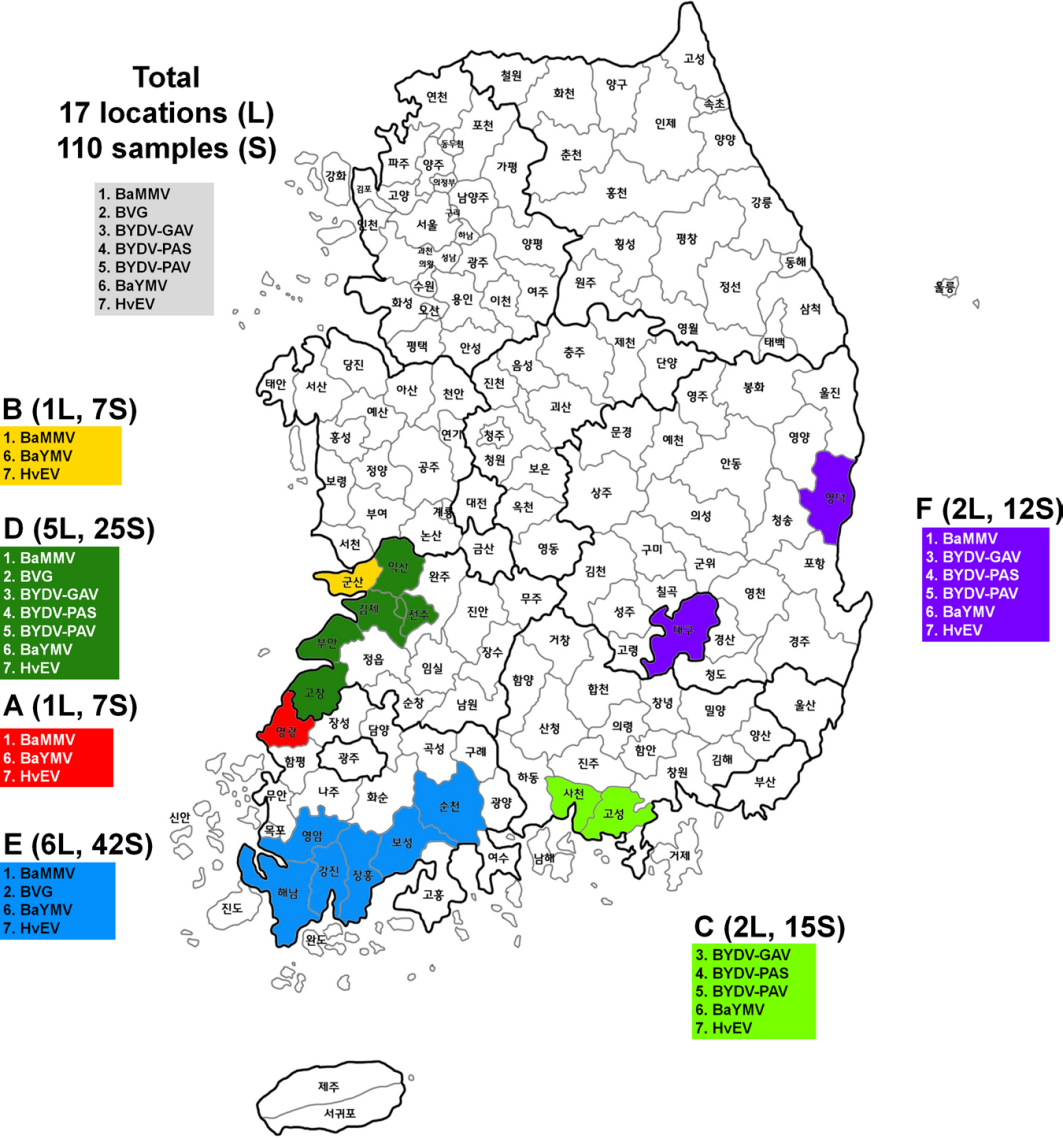
Geographical locations of collected barley samples in Korea and the list of identified viruses in each library. (**A**–**F**) with different colors indicate library names with corresponding number of sampled locations and samples. L and S indicate location and sample, respectively.

We examined the geographical distribution of identified viruses (Fig. 3). We found that the partial sequence of VcHV1 was derived from barley host gene. Therefore, VcHV1 was excluded for further analysis. In total, we identified five viruses infecting barley. The identified viruses in each library were diverse. For example, we identified three viruses, including BaMMV, BaYMV, and HvEV, from library A, while BYDV, BaYMV, and HvEV were identified from library C. BaYMV and HvEV, identified from all six libraries, were the most common viruses infecting barley, followed by BaMMV (identified from five libraries). BVG was identified from libraries D and E, whereas BYDV was identified from libraries C, D, F.

### Calculation of virus accumulation in each library

**Figure 4:**
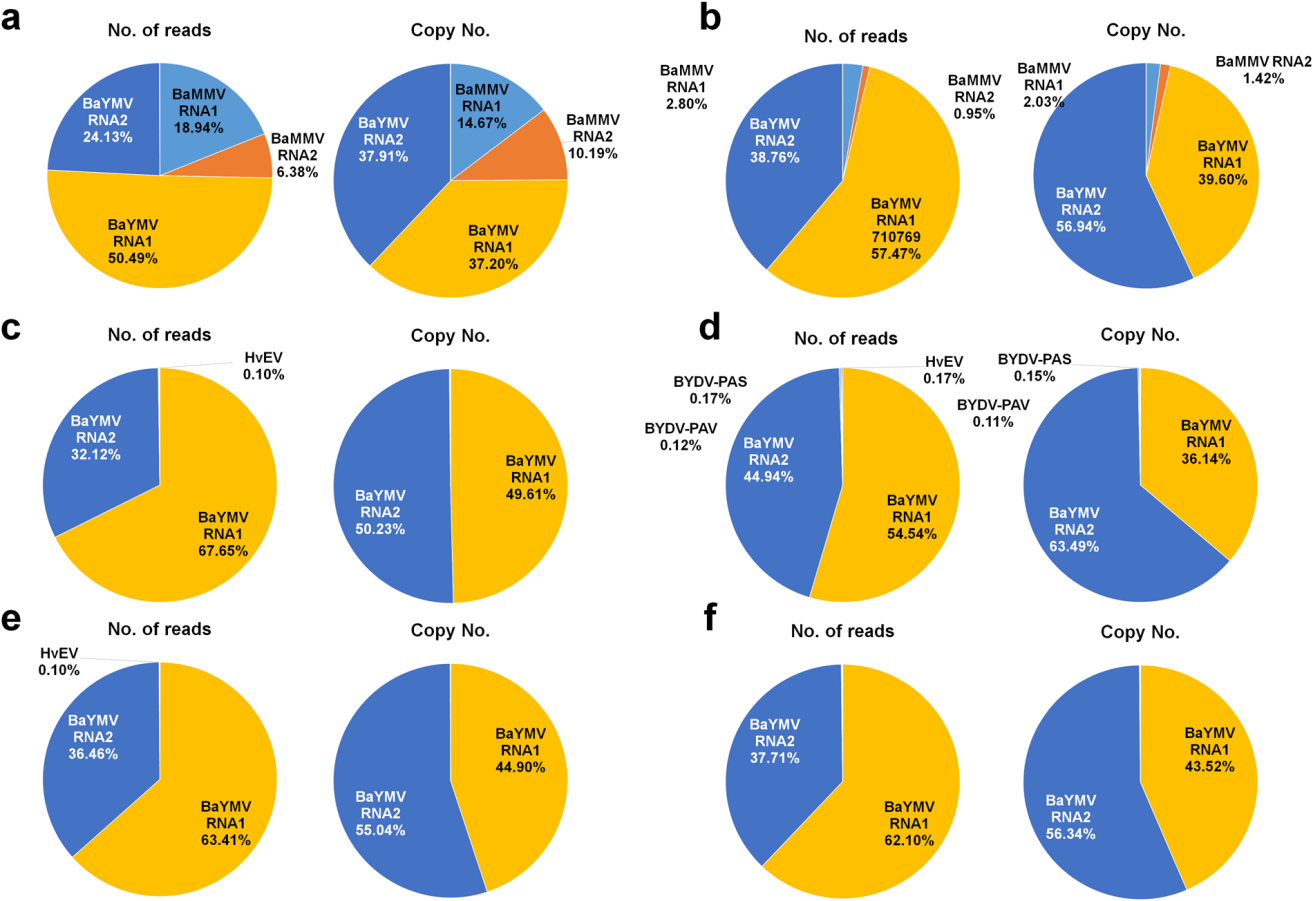
The proportion of identified viruses based on the number of virus-associated reads and copy number. Proportion of identified viruses for library A (**a**) library B (**b**) library C (**c**) library D (**d**) library E (**e**) and library F (**f**) were calculated based on number of reads and copy numbers.

We calculated virus accumulation in each library based on read number and copy number. Copy number was calculated by total read number divided by each viral genome size (Fig. 4). In all libraries, BaYMV was the dominant virus based on read number and copy number. Size of BaYMV RNA1 is greater than that of BaYMV RNA2. Therefore, the read number of BaYMV RNA1 is higher than that of BaYMV RNA2. However, based on copy number, the proportions of BaYMV RNA1 and RNA2 were very similar in libraries A and C. Furthermore, the proportion of BaMMV RNA1 and RNA2 was very similar in library A.

### *De novo* genome assembly and calculation of mutation rates

**Figure 5:**
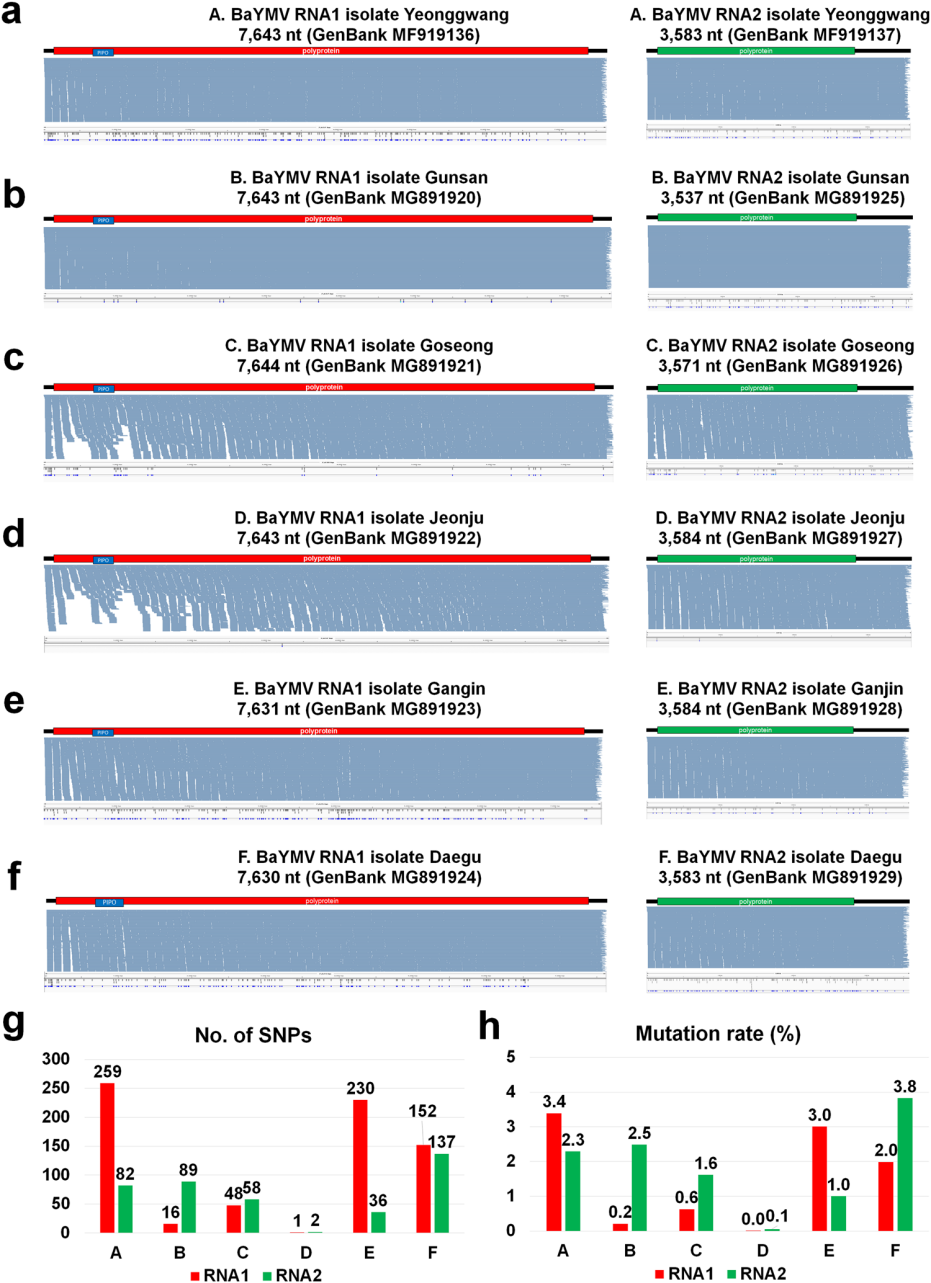
Identification of single nucleotide polymorphisms (SNPs) for BaYMV in six libraries. The genome organization, alignment of sequenced reads on BaYMV genome, and identified SNPs for BaYMV isolates Yeonggwang (**a**) Gusan (**b**) Goseong (**c**) Jeonju (**d**) Gangin (**e**) and Daegu (**f**). Blue bars indicate the position of identified SNPs on the BaYMV genome. The size of assembled BaYMV genome with respective accession number for individual BaYMV isolates was indicated. The number of identified SNPs (**g**) and the mutation rate (**h**) for each BaYMV isolate. RNA1 and RNA2 are indicated by red and green colors, respectively. The number of total SNPs was divided by the size of given virus genome for calculation of mutation rate.

**Figure 6:**
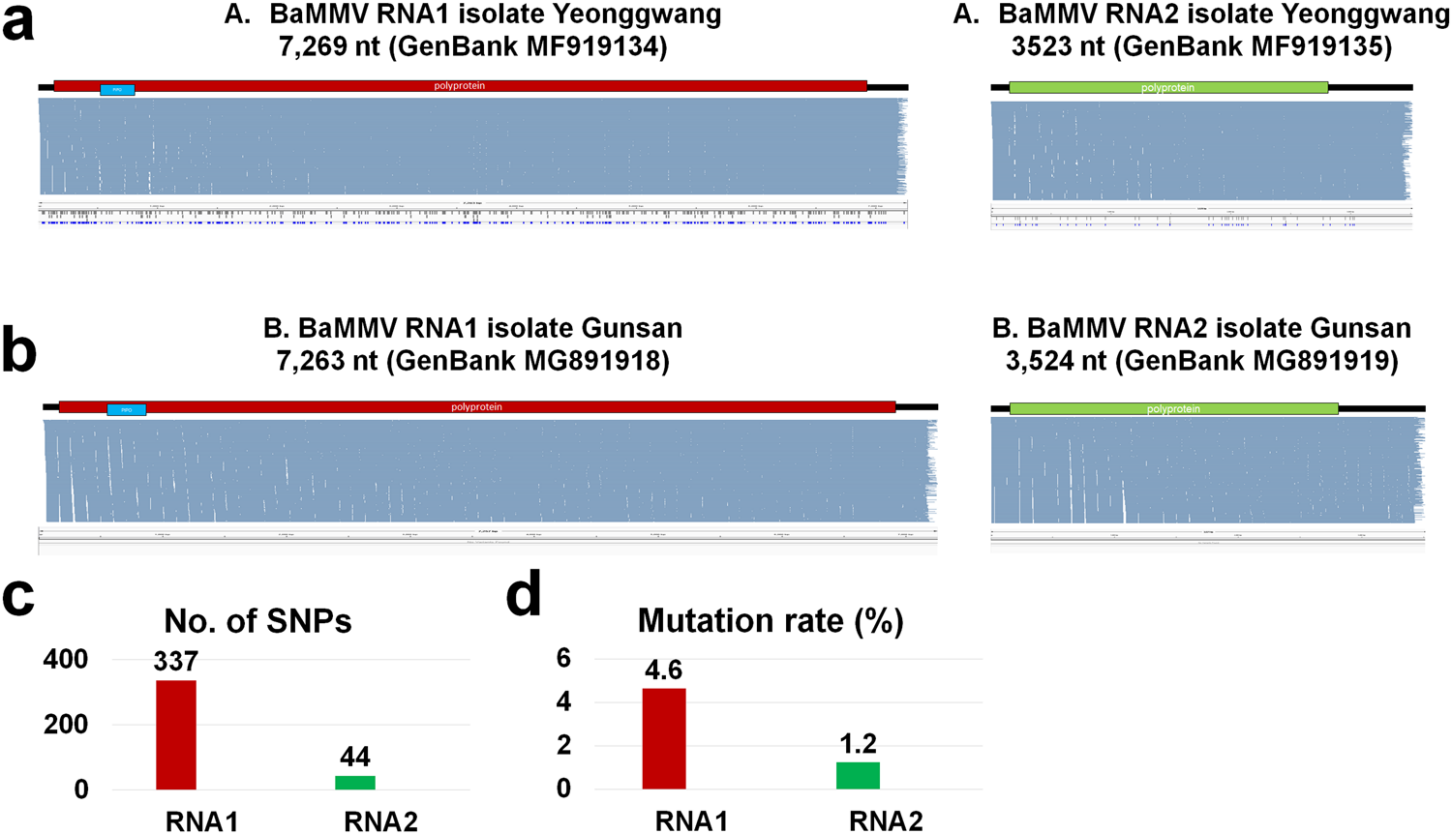
Identification of single nucleotide polymorphisms (SNPs) for BaMMV in A and B libraries. The genome organization, alignment of sequenced reads on BaMMV genome, and identified SNPs for BaMMV isolates Yeonggwang (**a**) and Gusan (**b**). Blue bars indicate the position of identified SNPs on the BaMMV genome. The size of assembled BaYMV genome with respective accession numbers for individual BaYMV isolates was indicated. The number of identified SNPs (g) and the mutation rate (h) for BaYMV isolate Yeonggwang. The BaMMV isolate Gunsan did not contain any SNP. RNA1 and RNA2 were indicated by red and green colors, respectively.

Using virus-associated contigs, we obtained six nearly complete genomes of BaYMV from six libraries (Fig. 5) and two BaMMV from libraries A and B (Fig. 6). Both viruses are composed of two RNA segments. The sizes of assembled BaYMV RNA1 ranged from 7,630 nucleotides (nt) (library F) to 7,644 nt (library C), while the size of assembled BaYMV RNA2 ranged from 3,537 nt (library B) to 3,584 nt (libraries D and E) (Fig. 5a–f). In order to analyze the SNP of each identified virus within each library, we mapped raw sequence reads on the assembled RNA genome. As shown in Fig. 5a–f, the number of reads mapped on each virus genome was sufficient to cover the nearly complete viral genome. We identified SNPs of BaYMV in each library (Fig. 5g). The numbers of identified SNPs were diverse among six libraries, for example ranging from three (library D) to 341 (library A) for BaYMV composed of two RNA segments. In case of BaYMV in libraries A and E, the number of identified SNPs for RNA1 was much higher than that for RNA2. However, the difference in identified SNP number between RNA1 and RNA2 was not significant for BaYMV in libraries C and F. Mutation rates of BaYMV ranged from 0.01 to 3.8 (Fig. 5h).

The sizes of assembled BaMMV RNA1 were 7,269 (library A) and 7,263 nt (library B), whereas the sizes of assembled BaMMV RNA2 were 3,523 nt (library A) and 3,524 nt (library B) (Fig. 6a,b). Interestingly, SNPs of BaMMV were only identified from library A while there was no SNP in BaMMV from library B. The numbers of SNPs for BaMMV from library A were 337 (BaMMV RNA1) and 44 (BaMMV RNA2), and their mutation rates were 4.6% and 1.2%, respectively.

### Phylogenetic relationships of BaYMV and BaMMV

**Figure 7:**
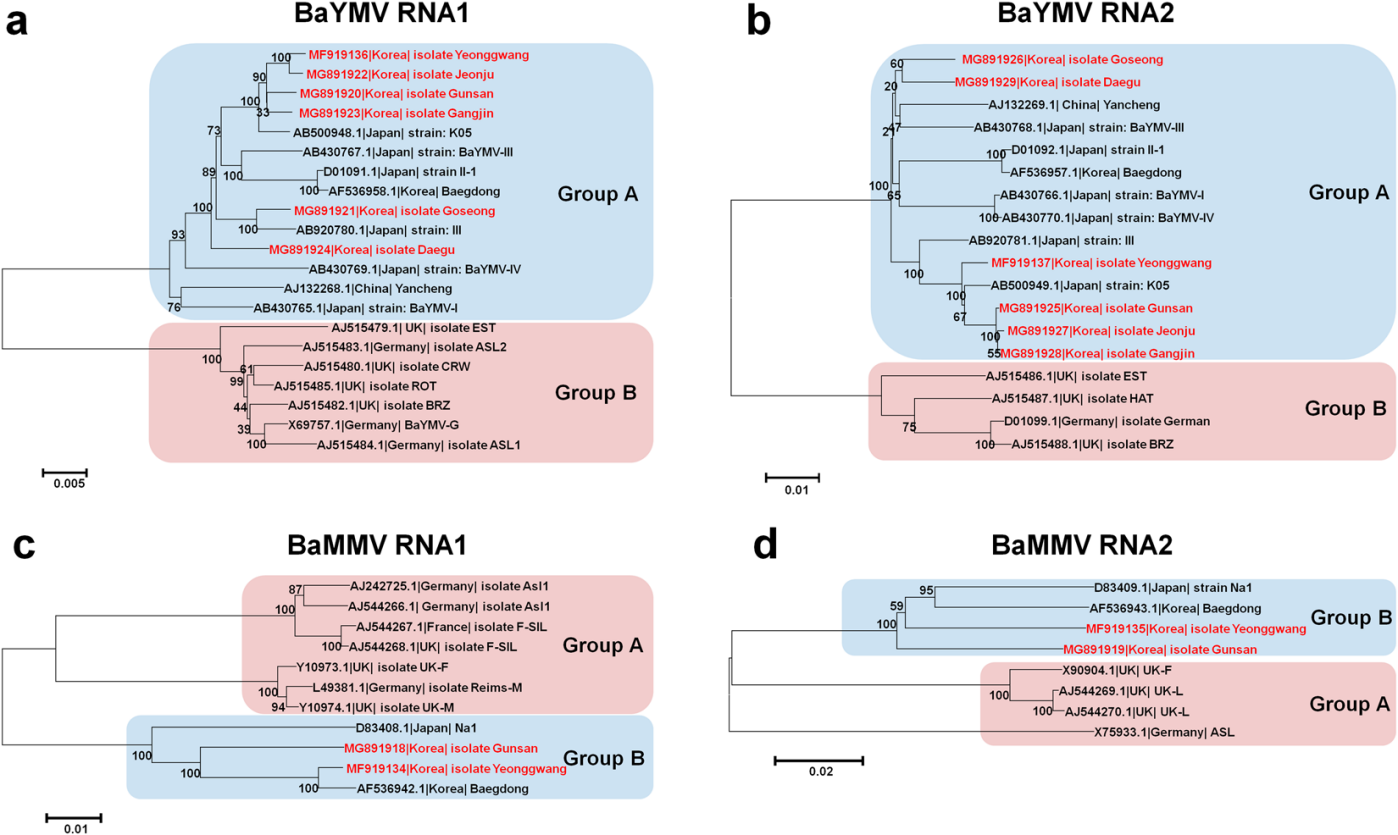
Phylogenetic relationship of the six BaYMV isolates and two BaMMV isolates identified. Phylogenetic relationships for six BaYMV isolates, Yeonggwang, Gunsan, Goseong, Jeonju, Gangin, and Daegu, based on assembled RNA1 (**a**) and RNA2 (**b**) sequences. Phylogenetic relationships for two BaMMV isolates, Yeonggwang and Gunsan, based on assembled RNA1 (**a**) and RNA2 (**b**) sequences. Available genome sequences for BaYMV and BaMMV were also used for phylogenetic construction. Accession number, country, and name of virus isolates or strains were also provided.

Based on assembled genome sequences for BaYMV and BaMMV, we generated phylogenetic trees. Since each virus is composed of two RNA fragments, two independent phylogenetic trees for each virus were generated (Fig. 7). The two phylogenetic trees using BaYMV RNA1 and RNA2 sequences showed that all six BaYMV isolates from this study were included in group A (Fig. 7a,b). Based on BaYMV RNA1 and RNA2 sequences, four isolates from libraries A, B, D, and E were grouped in the same clade with BaYMV isolate K05 from Kurashiki in Japan. The two isolates Goseong and Daegu, from libraries C and F, respectively, were closely related. In addition, BaYMV isolate Goseong showed sequence similarity to BaYMV strain III from Tochigi in Japan based on BaYMV RNA1 sequences. Interestingly, group A contains BaYMV isolates from three countries, China, Japan, and Korea, while group B contains BaYMV isolates from the United Kingdom and Germany.

Phylogenetic trees based on BaMMV RNA1 and RNA2 sequences also displayed two different groups of BaMMV isolates (Fig. 7c,d). The two isolates Gunsan and Yeonggwang were clustered together in group B. Group A again contains BaMMV isolates from three countries, Germany, France, and the United Kingdom, while Group B includes BaMMV isolates from Japan and Korea.

### Confirmation of NGS results by RT-PCR

**Figure 8:**
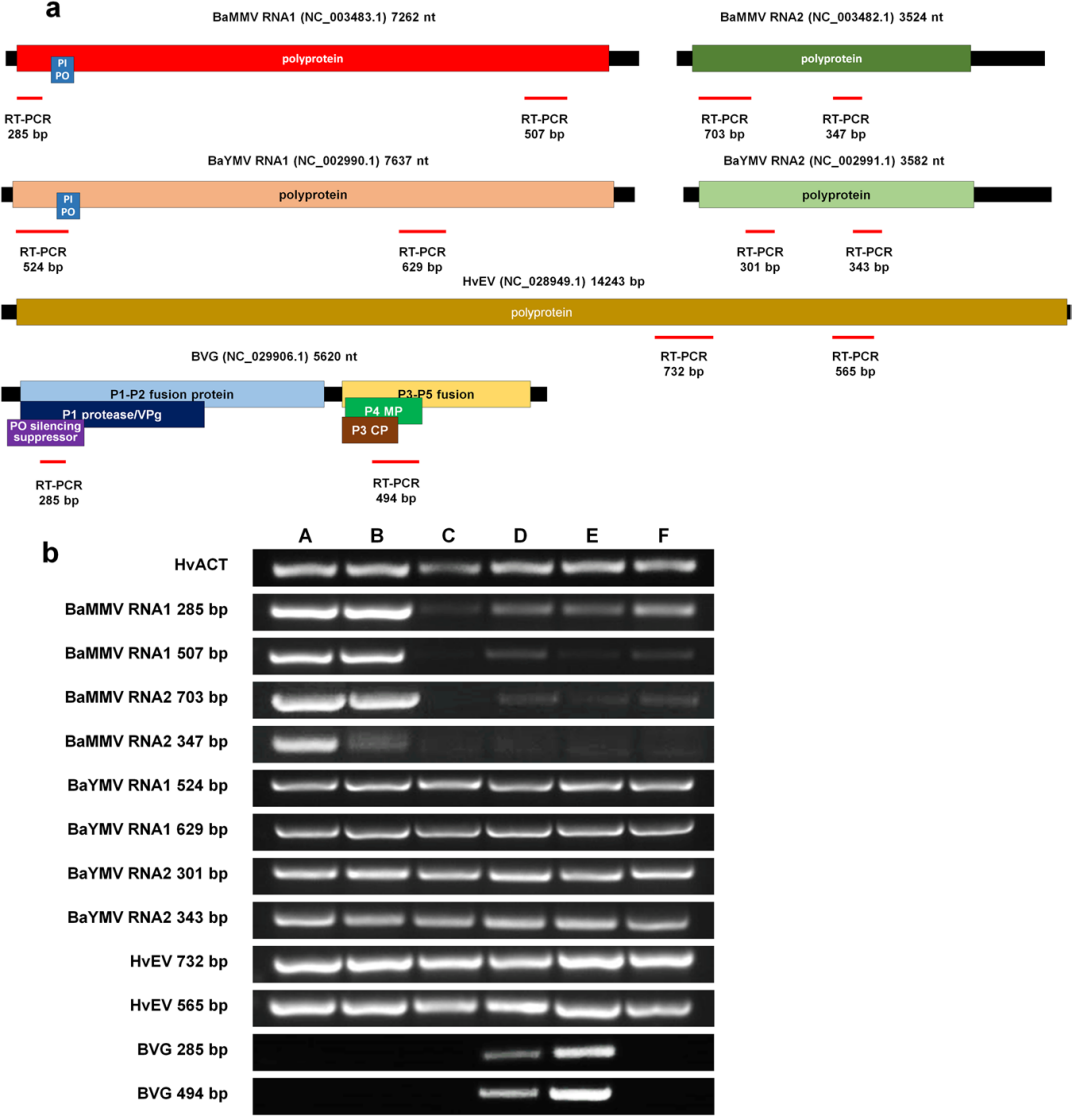
Confirmation of the identified four viruses by RT-PCR. (**a**) Primer-pairs for RT-PCR were newly designed based on obtained sequences for four viruses. The positions for amplicons with red bars are indicated on each virus genome organization. (**b**) RT-PCR results with virus specific primers. The barley actin gene was used as a positive control. The same total RNAs used for NGS were used for RT-PCR. Full length gels of RT-PCR results can be found in Fig. S1 in the supplementary information.

In order to confirm infection of identified viruses by NGS, we conducted RT-PCR using newly designed primers. To increase reliability of RT-PCR, we designed at least two different primer-pairs for each RNA fragment of four viruses, BaYMV, BaMMV, HvEV, and BVG (Fig. 8a and Table S2). Using four different primer-pairs for BaYMV, RT-PCR results clearly showed that all six libraries contained BaYMV sequences (Fig. 8b). In case of BaMMV, 285-bp PCR products were amplified in all six libraries, while 507-bp and 703-bp PCR products were not amplified from the library C. Furthermore, very weak bands were detected in libraries D, E, and F using primer-pairs amplifying 507-bp, 703-bp, and 347-bp of BaMMV sequences. Two primer-pairs of HvEV successfully amplified two PCR products (732-bp and 565-bp) from all six libraries. In addition, RT-PCR results showed that BVG was identified only from libraries D and E.

## Discussion

Recently, several NGS-based studies have been conducted for viruses infecting barley. For instance, dsRNA extraction followed by NGS was used to determine the complete genome sequences of HvEV, which is composed of dsRNA^23^. A recent study has examined genetic diversity of bymoviruses infecting barley in France using NGS and Sanger-sequencing, revealing that BaYMV-2 was responsible for the symptoms observed in varieties carrying the resistance gene *rym4*.

As compared to the previous studies associated with viruses infecting barley, our study focuses on barley RNA viromes associated with identification of viruses infecting barley, as well as geographical distribution and genetic diversity of identified viruses in Korea. We successfully identified several viruses (BaYMV, BaMMV, BYDV, BVG, and HvEV) by NGS followed by bioinformatics analyses. In addition, our study found that there were at least three different serotypes of BYDV infecting barley in Korea. Infection of BYDV strains PAV and MAV in barley and wheat have been reported in Korea; however, BYDV strains GAV and PAS infecting barley have not been reported in Korea. Moreover, we identified HvEV infecting barley in Korea for the first time. Prior to conducting barley virome study, we examined infection of three major viruses, BaYMV, BaMMV, and *Soil-borne wheat mosaic virus* (SBWMV), by RT-PCR using individual barley samples collected from the same major winter barley cultivation regions^27^. However, we did not identify SBWMV in our study. Moreover, BSMV was not identified in our study. BSMV has been identified in a wide range of areas in the world, including North Africa, North America, Europe, and Asia^28^. Thus, it is necessary to prevent the introduction of BSMV to avoid causing a serious loss of barley yield in Korea.

Although NGS is now generally used in many research areas, however, the price of NGS is still high for several individual samples in parallel. For the virome study of fruit trees that are mechanically infected by viruses, the library preparation using individual fruit trees followed by NGS is useful to decipher the viromes of individual fruit cultivars^29^. On the other hand, seed propagated plants are usually transmitted by insect vectors; therefore, it is efficient to pool samples for the identification of viruses infecting a plant species in different geographical regions. Accordingly, 110 samples from 17 geographical regions were pooled according to six different provinces. As we expected, each library contains a different list of viruses infecting barley, suggesting that geographical region is an important factor for the composition of viruses in each virome. However, several problems can be generated by pooling of samples which is a cost-effective approach in the virome study. For example, as discussed previously^30^, the misalignment of sequence reads could have occurred due to the presence of different virus isolates and the improper reference genome, although we used a consensus genome sequence assembled from NGS data. In fact, NGS is an effective method for identifying viral recombination^31^. However, the pooling of samples might interfere with the correct interpretation of recombination events due to a possible mixture of multiple virus isolates. Thus, our results might contain such problems.

There is still a dispute over the optimal method for plant virome study. A recent study has compared two different libraries using as small RNAs and ribosomal RNA-depleted total RNA, respectively, for NGS-based virus identification in plants^20^. Based on their results, the yield of viral sequences was dependent on each viral genome organization. In our study, we prepared cDNA libraries for NGS using oligo-dT primers in order to facilitate identification of two bymoviruses containing poly-A tail: BaYMV and BaMMV. However, it was not surprising that viruses composed of dsRNAs can be also be identified by mRNA libraries, as shown in other previous studies^32,33^.

Viruses most commonly infecting barley were BaYMV and HvEV, while other viruses including BaMMV, BYDV, and BVG were identified in specific libraries. Based on read number and copy number associated with identified viruses, BaYMV was the dominant virus in all six libraries. This result suggests that BaYMV could be a major virus in barley grown in Korea, which might be associated with yellow mosaic symptoms. Similarly, our previous study using RT-PCR also showed a high infection rate of single BaYMV in barley in Korea^27^. Furthermore, double infection of BaYMV and BaMMV was also confirmed in the barley samples of libraries A and E by NGS, as shown in the previous study by RT-PCR^27^. In particular, RT-PCR confirmed that all seven barley samples in Yeonggwang (library A) were co-infected by BaYMV, BaMMV, and HvEV (data not shown). HvEV is vertically transmitted by pollen and ovule infection^34^ and appears to be a non-pathogenic virus like other known endornaviruses that infect plants. Thus, the infection rate of HvEV in cultivated barley may be very high due to vertical transmission via seeds.

For four viruses, we conducted RT-PCR with two independent primer-pairs. Read numbers and RT-PCR results for each virus give the level of virus accumulation in each library. Except for HvEV, read number from NGS was correlated with band intensity from RT-PCR. In case of HvEV, the read number from NGS was low in all six libraries; however, the intensity of amplified RT-PCR products was high. Since HvEV is composed of dsRNA, it cannot be well recovered by an mRNA library using oligo-dT primer followed by NGS. Therefore, the quantification of virus accumulation should be carefully assessed by a combination of several methods.

Size of RNA fragment was an important factor to estimate virus accumulation by NGS. In general, a large number of virus-associated reads could be obtained from a large genome fragment of a virus. In case of BaYMV and BaMMV, which each consist of two RNA fragments, the number of reads from RNA1 was always higher than that from RNA2, since it is bigger than RNA1. In contrast, the copy number of BaYMV RNA2 was slightly higher than that of BaYMV RNA1, although we hypothesized that the copy numbers for RNA1 and RNA2 might be equal.

Due to the presence of poly-A tail in two bymoviruses, reads associated with two viruses were high enough to assemble two genomes *de novo*. Six BaYMV genomes and two BaMMV genomes were assembled by NGS followed by two *de novo* genome assemblers, Trinity and Velvet. The advantages and disadvantage for these two assemblers for virome study have previously been described^32^. Using assembled genomes, we studied phylogenetic relationships of BaYMV and BaMMV. Phylogenetic trees showed two distinct groups, which were divided by geographical region, Europe and Far East Asia. Historically, many barley plants in Korea have been collected by the Japanese during Japanese occupation, and many of them have been reintroduced to Korea^35^. Therefore, all Korean BaYMV and BaMMV isolates are in the same clade with Japanese isolates. Among five BaYMV isolates, four isolates showed sequence similarity to the known Japan strain K05. The BaYMV strain K05 was used as a template for the construction of infectious cDNA clones of a BaYMV leading to yellow mosaic disease in winter barley^36^. Based on those results, we suppose that BaYMV infecting barley in Korea that is closely related to strain K05 could be a major virus causing yellow mosaic disease in winter barley in Korea. Moreover, two BaMMV isolates were grouped in the known Japanese BaMMV strain Na1, which showed pathogenicity in different barley cultivars^37^, suggesting their possible pathogenicity.

In our study, the collected samples were pooled for the library preparation. The advantages of pooling samples might be the reduction of NGS cost and increase in the possibility of identifying viruses at a time. However, the disadvantage of pooling samples is that we cannot obtain detailed information on the viruses infecting a single plant. In general, a single plant is also frequently infected by diverse viruses or different isolates of a virus. Although we examined the SNPs of an individual assembled virus in our study, the SNP information did not represent the individual virus, but a collection of several isolates due to pooling samples. For example, SNPs for BaYMV and BaMMV using NGS data showed high mutation rates because pooled samples might contain a mixture of diverse isolates/strains for two viruses. However, BaYMV isolate Jeonju and BaMMV isolate Gunsan exhibited a few and no SNPs, respectively, suggesting low levels of genetic variation. Although samples were pooled, the mutation rates for two viruses were not correlated with the number of pooled samples. Library D pooled from 25 samples exhibited three SNPs for BaYMV, whereas Library A pooled from seven samples and Library F pooled from 12 samples showed 351 and 289 SNPs, respectively, for BaYMV. We next examined the correlation between the number of SNPs and the number of collected regions. The number of SNPs for BaYMV in Libraries A (one region), E (six regions), and F (two regions) was very high, while that in Library D (five regions) was very low, suggesting no correlation between the mutation rates and the number of collected regions. In fact, virus mutation is highly dependent on virus type, host type, and environmental conditions^38^. Our previous study also showed that the mutation rate of a plant virus or a viroid varied in different plants^29^. Therefore, we carefully hypothesized that libraries showing high mutation rates for identified viruses in our study might contain barley samples possessing the viruses with high mutation rates. Moreover, it is likely that not all, but a few, barley samples in the same library contained the viruses with high mutation rates.

In summary, six barley RNA viromes in this study provide a comprehensive overview of viruses infecting winter barley in Korea. Although several viruses infecting barley have been identified, we found that BaYMV was the dominant virus associated with yellow mosaic disease symptoms in Korea. Furthermore, phylogenetic trees using assembled viral genomes suggest that geographical region is a main factor to group BaYMV and BaMMV isolates/strains. Moreover, SNP analyses for BaYMV and BaMMV revealed genetic variations of two viruses in different geographical regions.

## Methods

### Plant materials

We collected 110 winter barley leaf samples from 17 geographical regions in Korea in March 2016. The 17 regions are the main winter barley production areas in Korea. Most barley samples showed yellow mosaic disease symptoms; however, some barley samples did not show any symptoms. The 110 samples were pooled according to six different provinces in Korea. Detailed information about geographical regions and the six different provinces can be found in Table 2 and Fig. 3.

### Total RNA extraction and library preparation

Pooled barley leaf samples were frozen using liquid nitrogen and ground with a pestle and mortar. Total RNA was extracted using the RNeasy Plant Mini Kit (Qiagen, Hilden, Germany) according to manufacturer’s manuals. The quality and quantity of extracted total RNA were measured using an Agilent 2100 Bioanalyzer (Agilent, Santa Clara, CA) and gel electrophoresis. The extracted total RNAs were used for the library preparation for RNA sequencing using the NEBNext Ultra™ RNA Library Prep Kit for Illumina in accordance with the manufacturer’s instructions (NEB, Ipswich, Massachusetts, U.S.A.). In brief, we extracted mRNAs with poly-A tail using poly-T oligo-attached magnetic beads. The first strand of cDNA was synthesized by the purified mRNAs followed by a second strand of cDNA. After that, the adenylation of 3′ ends was conducted. Adapters were ligated, and PCR amplification was carried out to selectively enrich DNA fragments with adapters and amplify the amount of DNA in the library, respectively. The 2100 Bioanalyzer was used for quality control of the generated libraries (Agilent, Santa Clara, U.S.A.). The six prepared libraries were paired-end sequenced by Macrogen Co. (Seoul, South Korea) using the HiSeq2000 platform.

### Bioinformatic analyses to identify viruses in the assembled transcriptome

We used the same bioinformatics analyses to identify viruses in the assembled transcriptome as described previously^29^. In brief, two different methods, Trinity program (version 2.0.2, released 22^nd^ January 2015) with default parameters^39^ and Velvet/Oases assembler (version 0.2.08)^40^, were used for *de novo* transcriptome assembly. Assembled contigs in each transcriptome were subjected to MEGABLAST^41^ with a cut-off E-value of 1e^−6^ search against NCBI’s viral reference database downloaded from https://www.ncbi.nlm.nih.gov/genome/viruses/. Only virus-associated contigs were selected after deleting endogenous virus-like sequences.

### *De novo* genome assembly of BaYMV and BaMMV

Nearly complete genomes for six BaYMV and two BaMMV genomes were assembled based on contigs generated by Trinity and Velvet assemblers as previously described^29,32^. ClustalW program implemented in the MEGA7 program was used to align contigs on the assembled viral genomes^42^. Again, we aligned raw sequence reads on the assembled viral genome to confirm consensus sequences using a Burrows-Wheeler Aligner (BWA) program with default parameters^43^.

### Generation of phylogenetic trees for BaYMV and BaMMV

To generate phylogenetic trees for BaYMV and BaMMV, six BaYMV and two BaMMV genomes were used. Each virus is a bipartite virus composed of two RNA fragments. Therefore, four independent phylogenetic trees were constructed. BLASTN was conducted to find known BaYMV and BaMMV genome sequences using the assembled BaYMV and BaMMV RNA fragments as a query against GenBank (http://www.ncbi.nlm.nih.gov/genbank/). Each RNA fragment sequence for each virus was aligned using the ClustalW program with default parameters. In *de novo* transcriptome assembly, some virus-associated contigs include partial sequences derived from the host plant. Those sequences can be identified by BLASTN search and sequence alignment on the reference virus genomes. Those unnecessary sequences derived from the host plant and poly-A tails at the 5′ and 3′ regions were deleted. We manually edited aligned sequences. A phylogenetic tree was constructed using the MEGA7 program with the neighbor-joining method with 1,000 bootstrap replicates and Kimura 2-parameter distance^42^.

### Analyses of SNPs for BaYMV and BaMMV using transcriptome data

SNPs for six BaYMV and two BaMMV transcriptomes were analyzed as described previously^29^. In brief, the raw sequence reads from each transcriptome were aligned on the identified individual viral genome using the BWA program with default parameters. The assembled viral genome derived from each transcriptome was used for the reference to increase SNP specificity. According to our experience, the application of known viral reference genomes results in the identification of unexpected SNPs. The SAM files generated by the BWA program were converted into BAM files using SAMtools^44^. The sorted BAM files were used to generate the VCF file format using the mpileup function of SAMtools for SNP calling. Finally, we used BCFtools implemented in SAMtools to call SNPs. The positions of identified SNPs on each viral genome were visualized by the Tablet program^45^.

### Design of primer-pairs and RT-PCR

We designed primer-pairs for four viruses, BaYMV, BaMMV, HvEV, and BVG. For each virus, two independent primer-pairs were designed. In case of BaYMV and BaMMV, two different primer-pairs for each RNA fragment were used. The same total RNA from the pooled sample was used as a template in RT-PCR. RT-PCR was conducted using the DiaStar™ OneStep RT-PCR Kit (SolGent, Daejeon, Korea), and the cycling conditions were 50 °C for 30 min, 95 °C for 15 min followed by 30 cycles at 95 °C for 20 sec, 50 °C to 56 °C for 40 sec (the annealing temperature can be variable depending on Tm values of primers), and 72 °C for 1 min, with a final extension at 72 °C for 5 min. The amplified RT-PCR products were confirmed by gel electrophoresis followed by EtBr staining. The amplified RT-PCR product was cloned in the pGEM-T-Easy Vector (Promega, Wisconsin, US) followed by Sanger sequencing.

## Electronic supplementary material

Supporting information

Table S1

Table S2

## Data Availability

The raw dataset in this study will be available, upon publication, in the Sequence Read Archive (SRA) repository with accession numbers SRR6706097, SRR6706098, SRR6706099, SRR6706100, SRR6706101, and SRR6706102. The six BaYMV and two BaMMV genome sequences obtained from this study were also deposited in GenBank, NCBI, with respective accession numbers.
