# Supplementary material for: Barley RNA viromes in six different geographical regions in Korea
Source: Sci Rep. 2018 Sep 5;8:13237. doi: 10.1038/s41598-018-31671-4 (PMC6125401; doi:10.1038/s41598-018-31671-4)

Supplementary Information

Barley RNA viromes in six different geographical regions in Korea

Yeonhwa Jo[1], Ju-Young Bae[2], Sang-Min Kim[2], Hoseong Choi[1], Bong Choon Lee[2,*], and Won Kyong Cho[1,*]

[1]Research Institute of Agriculture and Life Sciences, College of Agriculture and Life Sciences, Seoul National University, Seoul, 08826, Republic of Korea

[2]Crop Foundation Division, National Institute of Crop Science, RDA, Wanju, 55365, South Korea

Supplementary data

Supplementary Tables

Table S1. Summary of virus identification from six different barley transcriptomes.

Six different libraries, A–F, were sequenced. Assembled contigs by Trinity and Velvet assemblers, as well as raw sequence reads, were each blasted against the virus reference database. Trinity and Velvet indicate the number of assembled contigs associated with identified viruses. Sequence read indicates the number of raw sequence reads associated with identified viruses.

Table S2. Detailed information for the primers to detect viruses infecting barley used in this study.

Supplementary Figure

Figure S1. Full-length gels display RT-PCR results with virus-specific primers. The *actin* gene was used as a positive control.

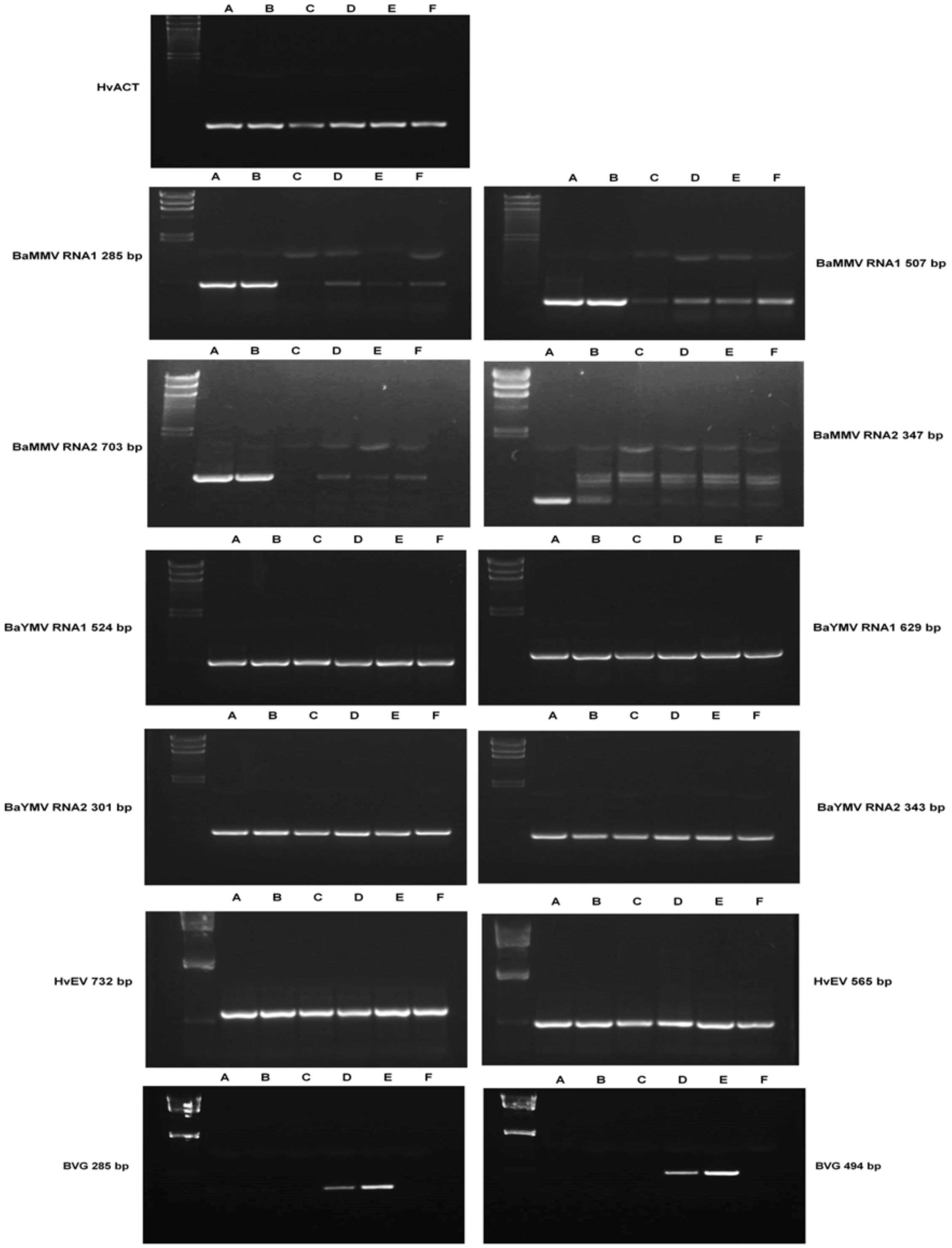

Supplement: Supplementary file 1 — Supporting information [file 41598_2018_31671_MOESM1_ESM.pdf]
